# Advances in Molecular Biology and Targeted Therapy of Mantle Cell Lymphoma

**DOI:** 10.3390/ijms20184417

**Published:** 2019-09-08

**Authors:** Pavel Klener

**Affiliations:** 1 First Dept. of Medicine-Hematology, General University Hospital in Prague, 128 08 Prague, Czech Republic; pavel.klener2@lf1.cuni.cz; 2 Institute of Pathological Physiology, First Faculty of Medicine, Charles University, 128 53 Prague, Czech Republic

**Keywords:** mantle cell lymphoma, cell cycle, B-cell receptor signaling

## Abstract

Mantle cell lymphoma (MCL) is a heterogeneous malignancy with a broad spectrum of clinical behavior from indolent to highly aggressive cases. Despite the fact that MCL remains in most cases incurable by currently applied immunochemotherapy, our increasing knowledge on the biology of MCL in the last two decades has led to the design, testing, and approval of several innovative agents that dramatically changed the treatment landscape for MCL patients. Most importantly, the implementation of new drugs and novel treatment algorithms into clinical practice has successfully translated into improved outcomes of MCL patients not only in the clinical trials, but also in real life. This review focuses on recent advances in our understanding of the pathogenesis of MCL, and provides a brief survey of currently used treatment options with special focus on mode of action of selected innovative anti-lymphoma molecules. Finally, it outlines future perspectives of patient management with progressive shift from generally applied immunotherapy toward risk-stratified, patient-tailored protocols that would implement innovative agents and/or procedures with the ultimate goal to eradicate the lymphoma and cure the patient.

## 1. Introduction

**Figure 1:**
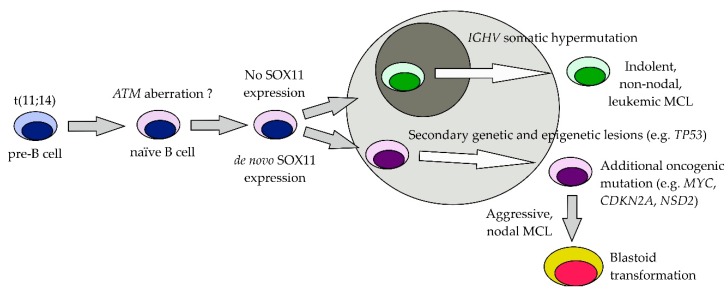
Pathogenesis of MCL. The Figure displays step-by-step accumulation of disease-critical mutations since early acquisition of t(11,14), *ATM* deletion, de novo expression of SOX11 (in nodal MCLs) to aggressive disease with *TP53* aberration, complex molecular–cytogenetic alterations or even complex karyotype changes.

Mantle cell lymphoma (MCL) accounts for approximately 7% of newly diagnosed non-Hodgkin lymphomas and in most instances is characterized by overexpression of cyclin D1 (CCND1) as a result of translocation t(11;14) (q13;q32). MCL is a heterogeneous disease with a broad spectrum of clinical behavior from indolent cases that do not require therapy for years to highly aggressive, hyperproliferative blastoid MCL [1]. It was repeatedly demonstrated that the clinical behavior directly or indirectly correlates with the genetic background of the disease. According to the WHO 2016 update of lymphoid malignancies, MCL now exists in two distinct categories (Figure 1) [2]. The first is nodal MCL (80–90% of cases) with unmutated immunoglobulin heavy chain variable region genes (*IGHV*), Sex-Determining Region Y-Box 11 (SOX11) overexpression, nodal and frequent extranodal involvement, and generally aggressive clinical behavior as a result of a higher degree of genomic instability. The cell of origin of the nodal MCL is believed to be a naïve, pre-germinal B-cell. The second is non-nodal leukemic MCL (10–20% of cases) with mutated *IGHV*, SOX11 negativity, lymphocytosis, splenomegaly, and typically indolent biological behavior due to low numbers of genetic lesions and epigenetic modifications. The cell of origin of the non-nodal leukemic MCL is presumably a memory B-cell with germinal center experience. Histologically, MCL can be divided into classical, pleomorphic, and blastoid morphology. MCL can also undergo histological transformation from classical to blastoid morphology, called blastoid transformation. In addition to this “classical” blastoid transformation, we have described and molecularly characterized the transformation from chronic lymphocytic leukemia (CLL) to blastoid MCL (MCL-variant Richter transformation) [3]. MCL affects more often men than women (2–3:1). The typical presentation at diagnosis includes generalized lymphadenomegaly (often in the form of bulky masses), splenomegaly (30–50%), bone marrow infiltration (70–80%), peripheral blood leukemization, and frequent extranodal (extramedullary) involvement (40–50%), typically of the gastrointestinal tract [4,5]. Central nervous system (CNS) involvement is detectable in <5% of patients at diagnosis, most frequently as leptomeningeal disease [6]. Diagnostic work-up includes lymph node and trephine biopsy with confirmation of overexpression of cyclin D1 and translocation t (11,14) by fluorescence in situ hybridization (FISH). Positron emission tomography–computed tomography (PET-CT) scan reveals 2-deoxy-2-fluoro-D-glucose (FDG)-avid lymphoma in a vast majority of cases. Flow cytometry usually confirms the presence of MCL clone with typical immunophenotype (CD20+, CD5+, CD22+, CD79b+, FMC-7+, CD23−, CD200−).

## 2. Pathogenesis of MCL

MCL cells are derived from antigen-experienced B lymphocytes [7,8]. Nodal and non-nodal MCLs are derived from different B-cell counterparts: germinal center (GC)-inexperienced naïve B-cell in the case of nodal MCL and GC-experienced memory B-cell in the case of non-nodal, leukemic MCL (Figure 1). The major factor that prevents naïve B-cells of the nodal MCLs to enter GC reactions is expression of sex-determining region Y-Box 11 (SOX11) neural transcription factor (see later).

### 2.1. Cyclin D1

Overexpression of cyclin D1 belongs to very early events in the process of oncogenic transformation. Apart from overexpression of full-length cyclin D1, a subset of hyperproliferative MCL was shown to harbor a truncated form of cyclin D1 in most cases as a result of genomic deletions in the *CCND1* 3′UTR region leading to transcription of short variants of cyclin D1 mRNA with increased stability [9]. In addition, cyclin D1 protein overexpression is further enhanced by its increased stabilization mediated by aberrant overactivation of the PI3K pathway [10]. Rare cases of cyclin D1-negative MCL are characterized by frequent rearrangements of *CCND2* and *CCND3* [11]. A subset of cyclin D1-/D2-/D3-negative MCL with aggressive features has cyclin E dysregulation [12].

### 2.2. Recurrent Molecular Cytogenetic Aberrations

Cyclin D1 overexpression alone is insufficient for malignant transformation of lymphocytes, which has been confirmed to require additional molecular aberrations [13,14,15]. Secondary genetic and epigenetic lesions leading to deregulation of key signaling pathways drive MCL pathogenesis. MCL represents a lymphoma subtype with high numbers of recurrent cytogenetic–molecular aberrations at diagnosis. Delfau-Larue et al. reported that as few as 20% of patients had no detectable copy number alteration besides the translocation t(11;14), while 80% of patients had one or more of the analyzed aberrations including deletions of tumor suppressor P53 (*TP53*), ataxia-telangiectasia mutated (*ATM*), cyclin-dependent kinase inhibitor 2A (*CDKN2A*), *CDKN1B*, and retinoblastoma 1 (*RB1*), or amplifications of B-cell lymphoma 2 (*BCL2*), V-Myc myelocytomatosis viral oncogene homolog (*MYC*), *CDK2*, *CDK4*, and human homolog of mouse double-minute 2 (*MDM2*).

In recent years, whole exome sequencing by next-generation sequencing approach enabled identification of recurrent somatic mutation in large numbers of patients at diagnosis and at disease relapse [13,14,16,17,18,19,20,21]. This enabled gaining insight into a complex interplay of genetic lesions and our better understanding of cell-intrinsic mechanisms that underlie lymphoma progression and drive drug resistance [22]. The most frequently mutated genes in MCL include *ATM* (40–50%), *CCND1* (14–35%), *TP53* (14–31%), mixed lineage leukemia protein 3 (*MLL3*, 16%), *MLL2* (12–20%), tumor necrosis factor associated factor 2 (*TRAF2*, 7–10%), *RB1* (10%), nuclear receptor binding SET domain protein 2 / Wolf-Hirschhorn syndrome candidate 1 (*NSD2/WHSC1*, 7–31%), baculoviral inhibitor of apoptosis (IAP) repeat containing 3 (*BIRC3*, 5–8%), *NOTCH1* (5–14%), *NOTCH2* (5%), *CDKN2A* (6%), and caspase recruitment domain family member 11 (*CARD11*).

## 3. Recurrent Molecular/Cytogenetic Lesions

Genetic lesions recurrently found in patients with newly diagnosed MCL can be grouped into several functional categories including cell cycle control (*CCND1*, *RB1*, *CDK2*, *CDK4*, *CDKN2A*, *CDKN1B*, *TP53*, *MYC*), genotoxic stress pathways (*TP53*, *ATM*, *CDKN2A*, *MDM2*), apoptosis (*BCL2*, *MDM2*, *TP53*, *CDKN2A*), key prosurvival cell signaling pathways (*TRAF2*, *BIRC3*, *CARD11*), and epigenetic regulation (*NSD2/WHSC1*, *MLL2*, *MLL3*, or SWI/SNF related, matrix associated, actin dependent regulator of chromatin *SMARCA4*) (Figure 1).

### 3.1. Genotoxic Stress Pathways

Deletions of 17p13 or mutations of *TP53* genes belong to the most frequent findings in MCL (20–34%) and were associated with poor outcome in the majority of studies published so far [23,24]. Interestingly, Eskelund et al. recently reported that *TP53* mutations correlated with significantly worse outcome compared to *TP53* deletions [25]. Immunohistochemistry (IHC) analysis of p53 protein expression correlated high p53 expression and lack of p53 expression with adverse outcome [26]. Curiously, lack of p53 protein expression did not correlate with biallelic *TP53* gene deletion and the reasons remain speculative. In a subset of MCL, TP53 inactivation can proceed through upregulation of MDM2 E3 ubiquitin-protein ligase.

Deletions of 9p lead to inactivation of *CDKN2A*, which encodes two different tumor suppressors: p16INK4A, an inhibitor of CDK4, and structurally unrelated p14ARF, transcribed by alternate open reading frame (ARF). P14ARF sequesters MDM2, which leads to p53 stabilization. Similarly to *TP53* alterations, *CDKN2A* deletions (monoallelic and biallelic) have been associated with adverse outcome in the majority of reports published so far, even in the context of high-dose cytarabine-based front-line therapies [23].

The ataxia-telangiectasia mutated (*ATM*) gene belongs to one of the most frequently deleted or mutated genes in newly diagnosed MCL patients (40–50%). Rarely, gains of *ATM* have been described too. *ATM* encodes a tumor suppressor involved in DNA damage response. Isolated *ATM* aberrations have never been associated with survival in MCL [19,23,27]. It was reported that *ATM*-deficient cells might be more susceptible to PARP1 inhibitors (e.g., olaparib, veliparib) and to radiotherapy [28,29]. To our best knowledge, these preclinical observations were unfortunately not validated in patients in clinical trials up to the present. Remarkably, *ATM* deletions have never correlated with inferior outcome for MCL. One plausible explanation is that *ATM* aberrations might on one hand increase genetic instability, but on the other hand might render lymphoma cells more sensitive to chemotherapy [30].

### 3.2. Cell Cycle Deregulation

**Figure 2:**
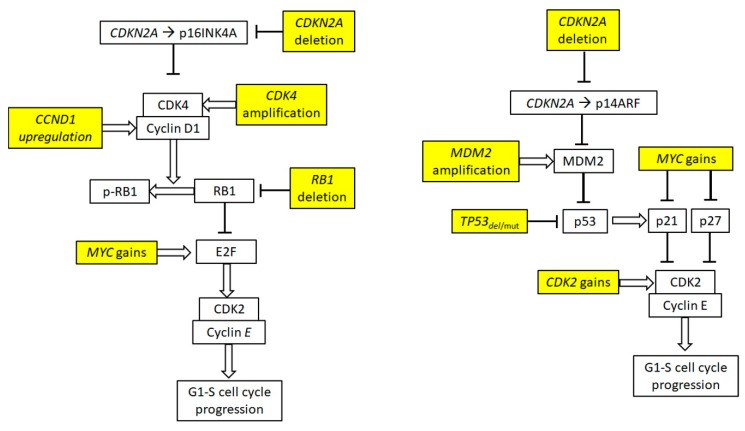
Cell cycle deregulation in MCL. MYC, MDM2, RB1, CCND1, CDKN2A.

Cell cycle deregulation is a hallmark of MCL. Overexpression of cyclin D1, amplification of *CDK4*, and deletion of *CDKN2A* synergistically enhance activity of the cyclin D1-CDK4 complexes, which mediate their oncogenic activity by sequestering a tumor suppressor retinoblastoma protein (RB1) by phosphorylation (Figure 2). RB1 protein inactivation by (hyper) phosphorylation (mediated by cyclin D1-CDK4 complexes) or by *RB1* gene deletion (observed in up to 30% of MCL) results in release of E2F transcription factor, a key trigger of G1-S phase transition [31]. E2F activity can be further boosted by *MYC* gains/amplifications, which has been associated with highly aggressive blastoid phenotype [32,33]. E2F activity induces accumulation of cyclin E-CDK2 complexes, the activity of which is enhanced by frequent *CDK2* gene amplifications and functional blockage of CDK2 inhibitors p21 and p27.

### 3.3. Deregulation of Apoptosis

B-cell lymphoma 2 (BCL2) protein belongs to key antiapoptotic molecules with frequent overexpression across B-NHL subtypes. Virtually all MCL primary cells (over)express BCL2. Molecular mechanisms of BCL2 overexpression in MCL are complex and comprise *BCL2* genomic gains (24%) and BCL2 mRNA overexpression as a result of aberrant activation of prosurvival pathways (e.g., nuclear factor kappa B (NFκB)) or as a result of loss of its negative regulators (e.g., loss of micro RNA *miR-15/16* as a result of frequent *13q* deletions). In addition, post-translational deregulations might contribute to BCL2 protein stabilization (e.g., decreased BCL2 degradation as a consequence of FBXO10 deficiency) [34,35,36,37]. Overexpression of myeloid cell leukemia 1 (MCL1), another key antiapoptotic protein, has been reported in MCL [38]. Biallelic deletions of BCL2-like 11 (*BCL2L11/BIM*) have been described in some studies, but not confirmed in our own study [39,40,41,42].

### 3.4. Prosurvival Signaling Cascades in MCL

#### 3.4.1. B-Cell Receptor (BCR) Signaling

**Figure 3:**
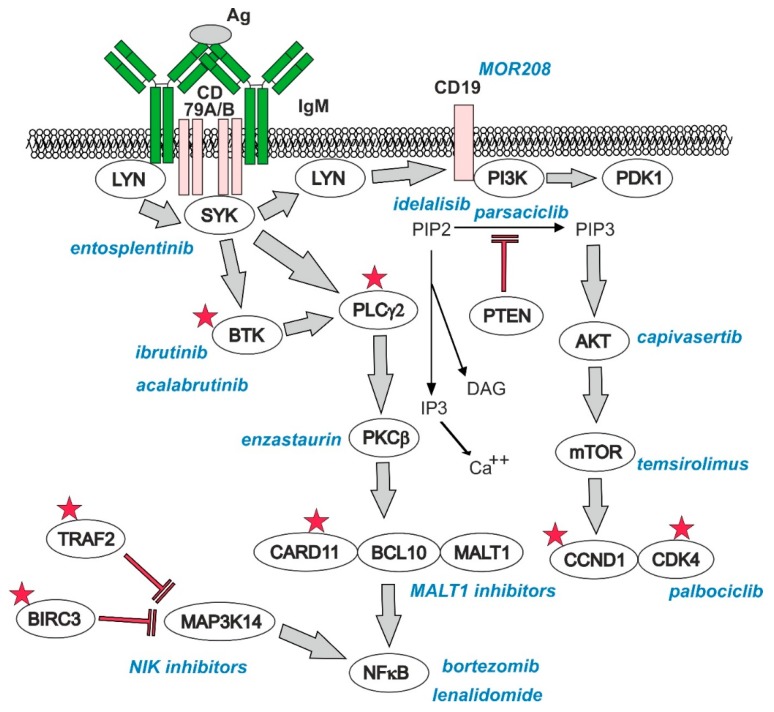
BCR signaling in MCL (asterisks indicate recurrently mutated genes in MCL; PIP2 = Phosphatidylinositol (4,5)-bisphosphate; PIP3 = phosphatidylinositol (3,4,5)-trisphosphate; DAG = diacylglycerole; PDK1 = phosphoinositide-dependent kinase 1).

BCR signaling plays a central role in the survival and proliferation of MCL cells (Figure 3) [43].

Indeed, the innovative anti-lymphoma drugs were designed to interfere with the aberrant BCR signaling to inhibit tumor proliferation and trigger apoptosis. BCR signaling leads to activation of the signalosome complex that triggers key downstream effector molecules, the aberrant activation of which orchestrates biology of MCL cells. At the same time, some of these molecules represent established or promising druggable targets in therapy of MCL including the spleen tyrosine kinase (SYK), Bruton’s tyrosine kinase (BTK), phosphoinositide-3 kinase (PI3K), protein kinase B (AKT), mammalian target of rapamycin (mTOR), nuclear factor kappa B (NFκB) transcription factors and their regulators, mucosa-associated lymphoid tissue lymphoma translocation protein (MALT1), and others. So-called chronic BCR signaling is activated by ligation of antigen to BCR and results in activation of BTK, phospholipase C gamma 2 (PLCγ2), protein kinase C (PKC), and CBM complex comprising caspase recruitment domain family member 11 (CARD11), B-cell lymphoma 10 (BCL10), and MALT1. So-called tonic BCR signaling is active even in the absence of antigen and signals predominantly through phosphoinositide 3-kinases (PI3Ks).

#### 3.4.2. PI3K–AKT–mTOR Pathway

PI3K is activated through BCR and CD19, as well as by oncogenic lesions, for example, overexpression of microRNA (miRNA) cluster miR-17–92 by chromosome 13q31-2 gains, which suppresses expression of PTEN and PHLPP2 phosphatases, key negative regulators of the PI3K–AKT–mTOR pathway [44]. A PI3K δ inhibitor, idelalisib, approved for the therapy of follicular lymphoma and chronic lymphocytic leukemia, demonstrated promising anti-lymphoma efficacy in R/R-MCL with 40% ORR, and a new generation PI3K δ inhibitor, parsaclisib, is currently tested in a phase II clinical trial in patients with R/R MCL (ClinicalTrials.gov number NCT03235544) [45].

#### 3.4.3. Nuclear Factor kappa B (NFκB) Pathway

BCR signaling leads to activation of the canonical NFκB pathway (BCR-NFκB), namely through the CARD11–BCL10–MALT1 (CBM) complex [46]. Upon CBM-mediated activation, the NFκB family of transcription factors (p65/RelA, c-Rel, RelB, p50/p105/NFκB1, and p52/p100/NFκB2) are released from their inhibitors belonging to the IκB family, and are translocated to the nucleus [47]. It was reported that ibrutinib-resistant MCL cell lines demonstrated genetic lesions leading to aberrant activation of the alternative NFκB pathway, namely activation of mitogen-activated protein kinase 14 (MAP3K14)/NFκB-inducing kinase (NIK). These genetic lesions comprise recurrent mutations of *TRAF2* and *BIRC3* in 6 and 10% of primary cell samples, respectively [18]. BIRC3/cIAP2 belongs to the family of inhibitors of apoptosis. Despite this denomination, BIRC3/cIAP2 is a poor inhibitor of caspases. Instead, BIRC3 functions as E3 ubiquitin ligase that regulates classical NFκB signaling [48]. Together with BIRC2/cIAP1, TRAF2, and TRAF3, BIRC3/cIAP2 forms a multiprotein complex that degrades MAP3K14/NIK kinase, thereby shutting down alternative NFκB pathway [49]. Loss-of-function mutations of *BIRC3*, *TRAF2*, and *TRAF3* thus result in aberrant overactivation of alternative NFκB signaling, which was repeatedly associated with drug resistance. Recurrent *BIRC3* mutations in patients with CLL were associated with resistance to fludarabine and independently correlated with inferior survival [50,51]. It was demonstrated that these genetic lesions conferred dependence of MCL cells on the protein kinase MAP3K14/NIK, which thus represents a promising druggable target in this subgroup of MCL. Another study reported recurrent mutations of *CARD11* (5.5% of 173 MCL samples) coding for a scaffold protein, an essential component of the CBM complex, which is required for BCR-induced NFκB activation in MCL primary cells [20]. By analogy with *TRAF2* and *BIRC3*, *CARD11* mutations conferred resistance to ibrutinib and to NFκB inhibitor lenalidomide [20]. MALT1, another key component of the CBM complex, was also reported to stabilize MYC oncoprotein, and its inhibition was associated with cytotoxicity in vitro and in vivo [52].

#### 3.4.4. Notch Pathway

During the canonical Notch signaling, ligands of the Delta-like (DLL1, 3, 4) and Jagged family (JAG1, JAG2) expressed on the surface of neighboring cells bind to the single-pass transmembrane Notch receptors (Notch1–4) on the target cells, thereby triggering γ-secretase-mediated cleavage of the intracellular part of Notch called INC that translocates to the nucleus, forming a short-lived multiprotein transcription factor complex [53]. C-terminal PEST (rich in proline (P), glutamic acid (E), serine (S), and threonine (T)) domain of INC is responsible for its rapid ubiquitin ligase-mediated degradation/inactivation. Like in CLL, *NOTCH* mutations recurrently found in 5–12% of MCL patients cluster mainly in the C-terminal PEST domain leading to enhanced stability of INC protein and aberrant (over)activation of Notch signaling, which is associated with shorter survival [17,54]. Therapeutic Notch targeting, however, remains so far a domain of preclinical research.

### 3.5. Epigenetic Modifiers in Pathogenesis of MCL

Mutations *NSD2* alias *WHSC1* (alias multiple myeloma SET domain-containing protein type III (*MMSET*)) coding for a histone methyltransferase specific for methylation of histone 3 lysine 36 (H3K36) results in reduced apoptosis and enhanced proliferation, clonogenicity, adhesion, and migration of the mutated cells [55]. It was reported that NSD2 mediates methylation of PTEN, thereby enhancing its ability to repair double-strand DNA breaks through dephosphorylation of yH2AX in the nucleus [56]. Inhibition of NSD2 sensitized cancer cells to PI3K inhibitors and DNA-damaging agents. In MCL, mutations of *NSD2*/*WHSC1* correlated with shorter survival and blastoid transformation [19,57]. *NSD2/WHSC1* thus emerged as a new relevant druggable target in MCL and other cancers with recurrent gene mutations [58].

The other epigenetic modifiers recurrently mutated in MCL comprise MLL2, MLL3, and SMARCA4. Loss-of-function mutations of *MLL2*, an H3K4 methyltransferase, has been described in diffuse large B-cell lymphoma [59]. Histone-deacetylase (HDAC) inhibitors belong to innovative anticancer drugs. Unfortunately, HDAC inhibitors tested so far (vorinostat, panobinostat, and abexinostat), single-agent or in combination with various anti-lymphoma drugs, demonstrated modest anti-MCL efficacy in clinical trials published so far [60,61,62,63,64]. Mutations in *SMARCA4* have been associated with poor response to ibrutinib plus venetoclax therapy through upregulation of BCL2L1/BCL-XL [65].

### 3.6. SOX11

As already mentioned, MCL can be divided into nodal and non-nodal, leukemic MCL [66]. Nodal MCL tends to be biologically aggressive and is characterized on a molecular level by unmutated status of the *IGHV* gene locus, and *de novo* expression of *SOX11* transcription factor, which is not expressed in normal B-cells. Non-nodal, leukemic MCL is typically an indolent disease with high levels of somatic hypermutation of IGHV and lack of SOX11 expression. A recent study identified differentially methylated regions in the *SOX11* promotor of nodal MCL cells, thereby providing at least partial explanation for the aberrant SOX11 expression in this MCL category [67]. SOX11 indeed has widespread impact on MCL biology. It contributes to tumor development by altering the terminal B-cell differentiation program and preventing MCL cells from entering germinal center reactions [68]. SOX11-mediated transactivation of PAX5 transcription factor leads to indirect blockage of plasma cell differentiation through suppression of PR/SET domain 1 (PRDM1), also known as B-lymphocyte-induced maturation protein 1 (BLIMP1) [69]. Overexpression of SOX11 in a transgenic mouse model (Eμ-SOX11-EGFP) led to enhanced BCR signaling in murine B-cells and induced oligoclonal B-cell hyperplasia in the spleen with an immunophenotype (CD5^+^CD19^+^CD23^−^) identical to human MCL [70]. In MCL cells, SOX11 regulates cell migration, invasion, growth, and angiogenesis [71,72].

In conclusion, pathogenesis of MCL probably proceeds over years with step-by-step accumulation of disease-critical mutations since early acquisition of t(11,14), de novo expression of SOX11 (in nodal MCLs) to aggressive disease with complex molecular–cytogenetic alterations (or even complex karyotype changes) (Figure 1). Not all patients are diagnosed with clinically manifest MCL, but rather, thanks to regular medical check-ups, based on detection of lymphocytosis during routine blood cell collections.

## 4. Prognostic Factors before Therapy

Mantle cell lymphoma prognostic index (MIPI) is a generally accepted, widely used, MCL-specific prognostic score based on four inputs: age, leukocytosis, lactate dehydrogenase (LDH), and performance status according to Eastern Cooperative Oncology Group (ECOG) [73,74]. MIPI can divide patients into three prognostic groups: high, intermediate, and low risk with five-year overall survival (OS) of 83%, 63%, and 34%, respectively [74]. Complex karyotype was repeatedly associated with dismal outcome [75,76]. Several important prognostic markers are derived from immunohistochemistry analysis of formalin-fixed, paraffin-embedded tissue sections. They include proliferation index by Ki-67, expression of SOX11, and morphology (classic versus pleomorphic/blastoid). In most published studies, Ki-67 ≥ 30% correlated with shorter survival [77,78]. A simple combination of MIPI and Ki-67 leads to so-called combined MIPI (MIPIc) that can divide patients into four prognostic groups with five-year OS of 85%, 72%, 43%, and 17% [78]. Blastoid variant MCL was associated with dismal outcome [79]. Absence of SOX11 expression was associated with non-nodal indolent forms of MCL (discussed above). The major disadvantage of these markers is their dependence on tissue biopsy. Not all patients are, however, necessarily subject to lymph node biopsy. Many patients with bone marrow infiltration can be diagnosed based on trephine biopsy, let alone the fact that patients with sufficient numbers of circulating MCL cells can be safely diagnosed based on flow cytometry and confirmation of t (11;14) translocation by locus-specific FISH.

Reliable prognostic markers that would enhance prognostic value of MIPI are needed for future proper stratification of front-line therapies. Many patients with low-risk disease benefit from a standard immunochemotherapy regimen (i.e., induction and maintenance therapy), or might be candidates for a diverse chemo-free regimen [80]. Patients with adverse prognostic factors (high-risk MIPI, Ki-67 ≥ 30%, TP53 and CDKN2A mutations, blastoid morphology) do not benefit even from the intensified immunochemotherapy regimen followed by autologous stem cell transplantation and maintenance.

Our better understanding of the pathophysiology of the disease will lead to more reliable prognostic (and predictive) markers, more efficient and less toxic treatment, improved outcome, and ultimately the eradication of MCL cells and curing of the patients.

## 5. Current Treatment Approaches and Outcomes of Patients after First-Line Treatment Approaches

Currently, the therapy of MCL is stratified basically by age (younger versus elderly), or more precisely, by the capability to undergo high-dose therapy (HDT) with autologous stem cell transplantation (ASCT) (i.e., transplantable versus nontransplantable patients). The physician’s decision, based on medical history, diagnostic work-up, and preferences of the patient, belongs to decisive factors in choosing the most suitable type of therapy [81]. For example, in the case of non-nodal MCL with no clinical symptoms that would indicate a need for therapy (e.g., B-symptoms, cytopenia, etc.), many patients are subject to watchful observation. In patients in the grey zone between younger and elderly (usually 65–70 years), adverse cytogenetics, blastoid morphology, high proliferation index by Ki-67, or extensive extranodal involvement might topple the decision-making in favor of an intensified approach with ASCT. Better stratification of upfront therapies with implementation of new targeted agents is an urgent task for the upcoming decade. At most centers, younger patients are treated with an intensified immunochemotherapy regimen based on anthracyclins, high-dose cytarabine, cisplatin, and anti-CD20 antibody (R)ituximab (alternation of R-CHOP, i.e., cyclophosphamide, vincristine, doxorubicin, and prednisone, and R-DHAP, i.e., cisplatin, high-dose cytarabine, and dexamethasone, Nordic MCL2 protocol, or MD Anderson protocol) [82,83,84,85]. Currently, the standard of care still implements consolidation in responders with HDT-ASCT, even if the benefit of HDT-ASCT is uncertain, especially in patients with TP53 aberrations [83,84,86]. All patients should be treated with rituximab maintenance (RM), usually every 2–3 months for 2–3 years [4,87,88,89,90]. The median OS of younger patients who finish the induction therapy and HDT-ASCT consolidation and start RM is more than 12 years [91,92]. Relapses in the low-risk MIPI patients are rare, indicating potential cure at least in some of these patients. Elderly patients are treated with an R-CHOP-like regimen or R-bendamustine, with or without cytarabine [85,93,94]. Bortezomib, in combination with cyclophosphamide, doxorubicin, and prednisone (so-called VR-CAP), was the only immunochemotherapy regimen that was associated with prolonged overall survival (OS) compared to R-CHOP in a phase III randomized trial [95]. Elderly patients treated with an R-CHOP-like regimen benefit from RM [5,96,97,98]. Maintenance therapies other than rituximab are currently tested in diverse clinical trials including lenalidomide or bortezomib [99,100]. Median progression-free survival (PFS) of the elderly patients who start RM is >5 years.

## 6. Prognostic Factors during and after Induction

Achievement of complete remission (CR) by CT or PET-CT has been repeatedly correlated with longer PFS [101]. Interestingly, time to progression is a strong, independent prognostic factor in patients with R/R MCL [102]. Minimal residual disease (MRD) is detection of a residual disease clone, which is not detectable by standard restaging procedures including CT, PET-CT, or trephine biopsy. It was repeatedly demonstrated that MRD status after induction therapy correlates with PFS [103]. Bone marrow is a better source for MRD detection compared to peripheral blood [101]. Reappearance of an MCL clone in a patient who had achieved MRD negativity is called “molecular relapse”, and it was demonstrated that molecular relapse precedes clinical relapse by several months. Regular monitoring of MRD in the follow-up period (i.e., after end of induction) thus enables identification of patients with molecular relapse of an MCL clone and subsequent therapeutic intervention, most commonly repeated administrations of anti-CD20 rituximab until MRD negativity has been restored [104]. In most centers, MRD is assessed by flow-cytometry (with sensitivity up to 10^−4^) or quantitative PCR (with sensitivity <10^−4^) [105]. Other targets for MRD detection (e.g., SOX11, cyclin D1 mRNA) are less commonly used [105]. Prognostic significance of MRD status after induction depends on the type of induction, as well as maintenance therapy. We have demonstrated that at least in the elderly patients treated with R-CHOP-like induction and rituximab maintenance, MRD status by quantitative PCR after induction loses its prognostic significance, most probably due to sustained immune-mediated control of the residual lymphoma clone (by rituximab) [101].

## 7. Salvage Therapy of Relapsed/Refractory MCL and Clonal Evolution of the Disease

Relapse/refractory (R/R) MCL is an incurable disease with median overall survival of 1–2 years [91]. Despite that, with our increasing knowledge on the biology of MCL achieved in the last two decades, several innovative agents have been designed, tested, and approved for the therapy of MCL, dramatically changing the treatment landscape for MCL patients. Most importantly, the improvements from clinical trials appear to have translated into real life [106,107]. Cell-intrinsic molecular mechanisms associated with relapse after first-line immunochemotherapy are still poorly understood. We have demonstrated that downregulation of deoxycytidine-kinase (dCK), the rate-limiting enzyme of the nucleotide salvage pathway, is responsible for acquired resistance not only to cytarabine, but also to other nucleoside analogues including fludarabine, gemcitabine, and cladribine [108]. In recent years, there have been emerging data on clonal evolution of MCL after failure of front-line therapies. Our better understanding of the molecular basis of the relapse will lead to better choices of the subsequent therapy, which is vital because, fortunately, there are many effective options now.

### 7.1. Bruton’s Tyrosine Kinase (BTK) Inhibitors

BTK inhibitors ibrutinib and acalabrutinib have revolutionized the therapy of R/R MCL. Ibrutinib and acalabrutinib were approved by the U.S. Food and Drug Administration (FDA) in 2013 and 2017, respectively [109,110]. Ibrutinib is currently the drug of choice for patients with R/R MCL who have undergone at least one systemic therapy [111]. In a phase 3 clinical trial, ibrutinib led to significant improvement in PFS and better tolerability compared to temsirolimus in patients with R/R MCL [112]. In prospective and retrospective studies published so far, ibrutinib monotherapy achieved an overall response rate (ORR) in 55–68% patients with median duration of response of 6–18 months [111,112,113,114,115]. Unfortunately, virtually all patients on ibrutinib sooner or later relapse. Relapses in patients who discontinue BTK inhibitors (for disease progression, blastoid transformation, or ibrutinib intolerance) represent an unmet medical need in the management of MCL patients. The majority of patients with relapses/progressions on ibrutinib have an especially dismal prognosis. The median OS after ibrutinib ranged between 3 and 10 months in most studies published so far [57,113,114,115]. No treatments that would improve the outcome of postibrutinib patients have been identified so far and this remains the major challenge for future clinical trials [113]. In transplant-eligible patients, ibrutinib bridging to allogeneic stem cell transplantation should be considered, especially in ibrutinib-sensitive R/R-MCL [116,117].

Molecular mechanisms of primary or acquired resistance to ibrutinib are a matter of extensive investigation [118,119]. In trials published so far, primary resistance to ibrutinib was observed in 10–35% patients. Mutations of *BTK*, for example, C481S, which alters the binding site of BTK from irreversible to reversible, leading to secondary ibrutinib resistance, have been detected only in a minority of ibrutinib-resistant MCL cells (15–20%). Aberrant activation of prosurvival signaling pathways, which substitute for the inhibited proximal BCR signaling, namely PI3K–AKT–mTOR, classical and alternative NFκB, or integrin-β1, was identified as a major player responsible for the acquired resistance to BTK inhibitors [118,120]. Molecular mechanisms underlying the reciprocal activation of prosurvival pathways are complex and are associated with both cell-intrinsic clonal evolution (selection of “favorable” somatic mutations) as well as cell-extrinsic dynamic feedback of MCL cells with the tumor microenvironment resulting in kinome adaptive reprogramming [121]. Mutation of *CARD11*, *MALT1*, *TRAF2*, *TRAF3*, or *BIRC3* lead to aberrant activation of classical or alternative NFκB [20]. In line with these observations, two of five patient-derived lymphoma xenografts used in our recent study on experimental therapy of MCL with the combination of venetoclax and MCL1 inhibitor S63845 harbored mutations of *TRAF2*, and both these PDX models were resistant to experimental therapy with ibrutinib (unpublished data) [42]. Enhanced dependence on BCL2 antiapoptotic signaling has been observed in ibrutinib-resistant cells. Concurrent mutations of *TP53* and *NSD2* were observed in three out of four patients with blastoid transformation on ibrutinib therapy. In a proportion of patients, relapses on ibrutinib tend to be especially aggressive. Aberrant activation of the PI3K–AKT–mTOR pathway might partially explain the aggressive phenotype of postibrutinib R/R MCL. In addition, Compagno et al. recently demonstrated that prolonged exposure to idelalisib, and to a lesser extent to ibrutinib, results in increased levels of activation-induced cytidine deaminase (AID), which results in enhanced genomic instability of malignant (and normal) B-cells. Increased AID expression could thus accelerate resistance to ibrutinib through increased mutational rate [122,123,124].

Rational combinations of ibrutinib and other targeted agents have shown promise in the therapy of R/R MCL including anti-CD20 rituximab, BCL2 inhibitor venetoclax, or CDK4 inhibitor palbociclib [125].

### 7.2. Bortezomib, Lenalidomide, Temsirolimus, and Bendamustine

Bortezomib was approved by the FDA in 2006 for therapy of R/R MCL [126]. The overall response rate after use of single-agent bortezomib reached 33% (8% CRs) with median PFS 9.2 months [127]. Bortezomib combinations with rituximab and either dexamethasone or bendamustine belong to options for R/R MCL [128,129,130].

Lenalidomide, another backbone antimyeloma drug, belongs to immunomodulatory agents. Lenalidomide was approved by the FDA for the therapy of R/R MCL in 2013. Several studies demonstrated efficacy of single-agent lenalidomide in patients with R/R MCL [131,132]. The overall response rate ranged from 26% to 40% with low rate of CRs and median PFS 4–9 months. The anti-lymphoma mode of action of lenalidomide appears to be mediated in large part by enhanced natural killer (NK) cell-mediated cytotoxicity via increased lytic immunological synapse formation and secretion of granzyme B [133].

Temsirolimus is an mTOR inhibitor approved for therapy of R/R MCL by European Medicines Agency (EMEA) in 2009. The overall response rate in studies published so far ranged from 22 to 40% with no CRs and median PFS 5–6 months [112,134].

Bendamustine is a “new-old” cytostatic agent originally developed in 1963 in Eastern Germany. In 2008 it was approved for the treatment of CLL. Bendamustine belongs to backbone cytostatic agents in the therapy of indolent and mantle cell lymphomas, as part of both front-line and salvage regimens, usually in combination with rituximab (R-B), with or without cytarabine (R-BAC) [93,94,97].

### 7.3. BCL2 Inhibitors

Venetoclax is a small-molecule, high-affinity BCL2 inhibitor that belongs to the family of BCL2 homology 3 (BH3) mimetics. Venetoclax displaces BH3-only proapoptotic proteins, like BCL2L11/BIM, from BCL2, thereby activating effector proapoptotic proteins BAX/BAK1 that disrupt mitochondria, thereby triggering apoptosis. Venetoclax demonstrated promising anti-lymphoma activity in R/R MCL including patients who discontinued ibrutinib [135,136]. Venetoclax is capable of inducing molecular remissions in a proportion of MCL patients. The combination of venetoclax and ibrutinib demonstrated safety and efficacy in a small clinical trial, and is currently being tested in a large randomized, placebo-controlled international phase 3 trail (NCT03112174) [125]. Another promising strategy is the combination of venetoclax and S63845, an inhibitor of MCL1, another key antiapoptotic molecule whose overexpression has been associated with venetoclax resistance [42].

### 7.4. PI3K Inhibitors and Inhibitors of Other Prosurvival Pathways

Based on increasing knowledge on molecular mechanisms associated with secondary ibrutinib resistance, PI3K inhibitors appear to belong to most promising class agents for the therapy of postibrutinib R/R MCL. The proof-of-concept study with idelalisib, the first-in-class PI3K δ inhibitor approved for the treatment of cancer (specifically, chronic lymphocytic leukemia and follicular lymphoma), demonstrated that targeting PI3K is a viable strategy in MCL [45]. Results from the phase 2 study with parsaciclib, a second-generation PI3Kδ inhibitor, in patients with R/R MCL are pending (ClinicalTrials.gov Identifier NCT03235544).

Inhibitors of spleen tyrosine kinase SYK (entosplentinib) and protein kinase C PKC (enzastaurin) demonstrated modest single-agent anti-lymphoma efficacy in R/R MCL, but either of these agents or second-generation inhibitors might in future prove effective as part of drug combinations or diverse maintenance therapies [137,138].

### 7.5. Immunotherapy Approaches in Experimental Therapy of R/R MCL

Immunotherapy already has an established place in treatment algorithms of MCL. Specifically, RM significantly prolongs survival of patients with MCL, in large part by engaging natural killer (NK) cell-mediated cytotoxicity. In analogy, lenalidomide mode of action also relies predominantly on NK cells. The combination of rituximab and lenalidomide (R^2^ regime) is a promising strategy both as a front-line therapy and as a maintenance [80].

Apart from NK-cell-based approaches, the recent introduction of T-cell-based immunotherapy approaches into clinical hemato-oncology, including immune checkpoint inhibitors (e.g., ipilimumab, nivolumab, or pembrolizumab) or bispecific T-cell engagers (e.g., blinatumomab), has revolutionized treatment of many types of solid tumors and hematologic malignancies including acute lymphoblastic leukemia or Hodgkin’s lymphoma. Unfortunately, MCL cells express very low levels of programmed cell death (PD) ligands (PDL1, PDL2), and almost no PD-1-expressing T-cells were found in MCL biopsies [139]. In consequence, unlike other types of cancer, the role for immune checkpoint inhibitors appears limited in the therapy of R/R MCL, at least from the perspective of current knowledge.

Genetically engineered T-cells that express chimeric antigen receptor (i.e., CAR T-cells) belong to adoptive T-cell approaches approved for the therapy of many malignancies including diffuse large B-cell lymphoma [140]. Results from the ZUMA-2 (NCT02601313) phase 2 study evaluating efficacy of autologous CD19 CAR T-cells (KTE-C19) in patients with R/R MCL are eagerly awaited.

Besides CAR T-cells, other T-cell-based immunotherapy approaches including adoptive T-cell-based strategies and dendritic cell (DC) vaccination have shown promise in experimental therapy of MCL, especially in eradication of minimal residual disease after successful debulking (by analogy with long-term clinical experience with allogenetic stem cell transplantation). These strategies include activation of cytotoxic T-cells generated with autologous dendritic cells conditioned with interferon (IFN-DC) and pulsed with immunogenic tumor cell lysates [141]. Importantly, the combination of both NK-cell- and T-cell-based approaches (e.g., rituximab and IFN-DC-based vaccination) might lead to antitumor synergy for more effective combination therapy of lymphoma patients [142].

## 8. Conclusions

**Figure 4:**
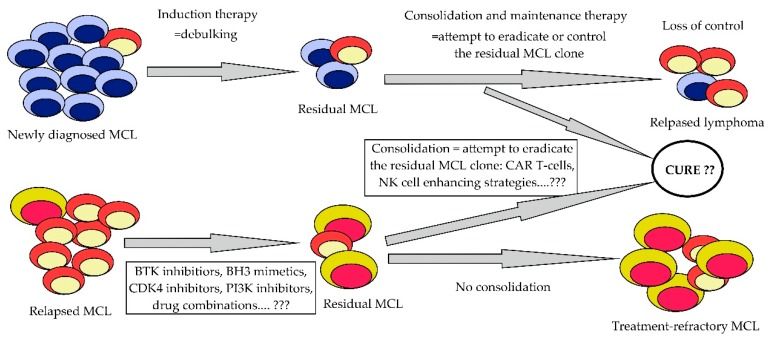
Current and future treatment algorithms in front-line and salvage therapy of MCL.

In the last decade, our knowledge on the biology and clonal evolution of MCL has significantly improved. New treatment algorithms based on intensified chemotherapy regimens that implemented high-dose cytarabine, platin derivatives, and rituximab maintenance led to better control of the disease in newly diagnosed patients, while the advent of many effective innovative molecules, including ibrutinib, lenalidomide, bendamustine, or venetoclax, have revolutionized the therapy of patients with relapsed/refractory disease. In the near future, the therapy of MCL will become risk-stratified and patient-tailored. New agents or novel rational drug combinations and treatment protocols will hopefully lead not only to better control of the disease, but also to the effective eradication of the residual MCL clone with permanent disease cure (Figure 4).

## Abbreviations

BCR	B-cell receptor
BTK	Bruton’s tyrosine kinase
CAR	Chimeric antigen receptor
CDK	Cyclin-dependent kinase
CR	Complete remission
HDT-ASCT	High-dose therapy–Autologous stem cell transplantation
MCL	Mantle cell lymphoma
MIPI	MCL international prognostic index
MRD	Minimal residual disease
NK	Natural killer (cells)
ORR	Overall response rate (= complete and partial remissions)
OS	Overall survival
PD	Programmed cell death
PFS	Progression-free survival
PI3K	Phosphoinositide-3 kinase
PD-L1/2	PD ligand 1/2
PR	Partial remission
R-CHOP	Rituximab + cyclophosphamide + doxorubicin + vincristine + prednisone
R-DHAP	Rituximab + dexamethasone + high-dose cytarabine + cisplatin
RM	Rituximab maintenance
R/R-MCL	Relapsed/refractory MCL

## References

[1] Cheah C.Y., Seymour J.F., Wang M.L. (2016). Mantle Cell Lymphoma. J. Clin. Oncol..

[2] Swerdlow S.H., Campo E., Pileri S.A., Harris N.L., Stein H., Siebert R., Advani R., Ghielmini M., Salles G.A., Zelenetz A.D. (2016). The 2016 revision of the World Health Organization classification of lymphoid neoplasms. Blood.

[3] Klener P., Fronkova E., Berkova A., Jaksa R., Lhotska H., Forsterova K., Soukup J., Kulvait V., Vargova J., Fiser K. (2016). Mantle cell lymphoma-variant Richter syndrome: Detailed molecular-cytogenetic and backtracking analysis reveals slow evolution of a pre-MCL clone in parallel with CLL over several years. Int. J. Cancer.

[4] Klener P., Salek D., Pytlik R., Mocikova H., Forsterova K., Blahovcova P., Campr V., Prochazka V., Obr A., Jaksa R. (2019). Rituximab maintenance significantly prolongs progression-free survival of patients with newly diagnosed mantle cell lymphoma treated with the Nordic MCL2 protocol and autologous stem cell transplantation. Am. J. Hematol..

[5] Klener P., Fronkova E., Belada D., Forsterova K., Pytlik R., Kalinova M., Simkovic M., Salek D., Mocikova H., Prochazka V. (2018). Alternating R-CHOP and R-cytarabine is a safe and effective regimen for transplant-ineligible patients with a newly diagnosed mantle cell lymphoma. Hematol. Oncol..

[6] Cheah C.Y., George A., Gine E., Chiappella A., Kluin-Nelemans H.C., Jurczak W., Krawczyk K., Mocikova H., Klener P., Salek D. (2013). Central nervous system involvement in mantle cell lymphoma: Clinical features, prognostic factors and outcomes from the European Mantle Cell Lymphoma Network. Ann. Oncol..

[7] Hadzidimitriou A., Agathangelidis A., Darzentas N., Murray F., Delfau-Larue M.H., Pedersen L.B., Lopez A.N., Dagklis A., Rombout P., Beldjord K. (2011). Is there a role for antigen selection in mantle cell lymphoma? Immunogenetic support from a series of 807 cases. Blood.

[8] Xochelli A., Sutton L.A., Agathangelidis A., Stalika E., Karypidou M., Marantidou F., Lopez A.N., Papadopoulos G., Supikova J., Groenen P. (2015). Molecular evidence for antigen drive in the natural history of mantle cell lymphoma. Am. J. Pathol..

[9] Wiestner A., Tehrani M., Chiorazzi M., Wright G., Gibellini F., Nakayama K., Liu H., Rosenwald A., Muller-Hermelink H.K., Ott G. (2007). Point mutations and genomic deletions in *CCND1* create stable truncated cyclin D1 mRNAs that are associated with increased proliferation rate and shorter survival. Blood.

[10] Dal Col J., Dolcetti R. (2008). GSK-3beta inhibition: At the crossroad between Akt and mTOR constitutive activation to enhance cyclin D1 protein stability in mantle cell lymphoma. Cell Cycle.

[11] Salaverria I., Royo C., Carvajal-Cuenca A., Clot G., Navarro A., Valera A., Song J.Y., Woroniecka R., Rymkiewicz G., Klapper W. (2013). CCND2 rearrangements are the most frequent genetic events in cyclin D1^−^ mantle cell lymphoma. Blood.

[12] Martin-Garcia D., Navarro A., Valdes-Mas R., Clot G., Gutierrez-Abril J., Prieto M., Ribera-Cortada I., Woroniecka R., Rymkiewicz G., Bens S. (2019). CCND2 and CCND3 hijack immunoglobulin light-chain enhancers in cyclin D1^−^ mantle cell lymphoma. Blood.

[13] Zhang J., Jima D., Moffitt A.B., Liu Q., Czader M., Hsi E.D., Fedoriw Y., Dunphy C.H., Richards K.L., Gill J.I. (2014). The genomic landscape of mantle cell lymphoma is related to the epigenetically determined chromatin state of normal B cells. Blood.

[14] Bea S., Valdes-Mas R., Navarro A., Salaverria I., Martin-Garcia D., Jares P., Gine E., Pinyol M., Royo C., Nadeu F. (2013). Landscape of somatic mutations and clonal evolution in mantle cell lymphoma. Proc. Natl. Acad. Sci. USA.

[15] Lovec H., Grzeschiczek A., Kowalski M.B., Moroy T. (1994). Cyclin D1/bcl-1 cooperates with myc genes in the generation of B-cell lymphoma in transgenic mice. EMBO J..

[16] Watson I.R., Takahashi K., Futreal P.A., Chin L. (2013). Emerging patterns of somatic mutations in cancer. Nat. Rev. Genet..

[17] Kridel R., Meissner B., Rogic S., Boyle M., Telenius A., Woolcock B., Gunawardana J., Jenkins C., Cochrane C., Ben-Neriah S. (2012). Whole transcriptome sequencing reveals recurrent NOTCH1 mutations in mantle cell lymphoma. Blood.

[18] Rahal R., Frick M., Romero R., Korn J.M., Kridel R., Chan F.C., Meissner B., Bhang H.E., Ruddy D., Kauffmann A. (2014). Pharmacological and genomic profiling identifies NF-kappaB-targeted treatment strategies for mantle cell lymphoma. Nat. Med..

[19] Yang P., Zhang W., Wang J., Liu Y., An R., Jing H. (2018). Genomic landscape and prognostic analysis of mantle cell lymphoma. Cancer Gene Ther..

[20] Wu C., de Miranda N.F., Chen L., Wasik A.M., Mansouri L., Jurczak W., Galazka K., Dlugosz-Danecka M., Machaczka M., Zhang H. (2016). Genetic heterogeneity in primary and relapsed mantle cell lymphomas: Impact of recurrent *CARD11* mutations. Oncotarget.

[21] Onaindia A., Medeiros L.J., Patel K.P. (2017). Clinical utility of recently identified diagnostic, prognostic, and predictive molecular biomarkers in mature B-cell neoplasms. Mod. Pathol..

[22] Ahmed M., Zhang L., Nomie K., Lam L., Wang M. (2016). Gene mutations and actionable genetic lesions in mantle cell lymphoma. Oncotarget.

[23] Delfau-Larue M.H., Klapper W., Berger F., Jardin F., Briere J., Salles G., Casasnovas O., Feugier P., Haioun C., Ribrag V. (2015). High-dose cytarabine does not overcome the adverse prognostic value of CDKN2A and TP53 deletions in mantle cell lymphoma. Blood.

[24] Greiner T.C., Moynihan M.J., Chan W.C., Lytle D.M., Pedersen A., Anderson J.R., Weisenburger D.D. (1996). p53 mutations in mantle cell lymphoma are associated with variant cytology and predict a poor prognosis. Blood.

[25] Eskelund C.W., Dahl C., Hansen J.W., Westman M., Kolstad A., Pedersen L.B., Montano-Almendras C.P., Husby S., Freiburghaus C., Ek S. (2017). TP53 mutations identify younger mantle cell lymphoma patients who do not benefit from intensive chemoimmunotherapy. Blood.

[26] Aukema S.M., Hoster E., Rosenwald A., Canoni D., Delfau-Larue M.H., Rymkiewicz G., Thorns C., Hartmann S., Kluin-Nelemans H., Hermine O. (2018). Expression of TP53 is associated with the outcome of MCL independent of MIPI and Ki-67 in trials of the European MCL Network. Blood.

[27] Greiner T.C., Dasgupta C., Ho V.V., Weisenburger D.D., Smith L.M., Lynch J.C., Vose J.M., Fu K., Armitage J.O., Braziel R.M. (2006). Mutation and genomic deletion status of ataxia telangiectasia mutated (ATM) and p53 confer specific gene expression profiles in mantle cell lymphoma. Proc. Natl. Acad. Sci. USA.

[28] Williamson C.T., Kubota E., Hamill J.D., Klimowicz A., Ye R., Muzik H., Dean M., Tu L., Gilley D., Magliocco A.M. (2012). Enhanced cytotoxicity of PARP inhibition in mantle cell lymphoma harbouring mutations in both ATM and p53. EMBO Mol. Med..

[29] Golla R.M., Li M., Shen Y., Ji M., Yan Y., Fu K., Greiner T.C., McKeithan T.W., Chan W.C. (2012). Inhibition of poly(ADP-ribose) polymerase (PARP) and ataxia telangiectasia mutated (ATM) on the chemosensitivity of mantle cell lymphoma to agents that induce DNA strand breaks. Hematol. Oncol..

[30] Yan W., Yang Y., Yang W. (2019). Inhibition of SKP2 Activity Impaired ATM-Mediated DNA Repair and Enhanced Sensitivity of Cisplatin-Resistant Mantle Cell Lymphoma Cells. Cancer Biother. Radiopharm..

[31] Jares P., Colomer D., Campo E. (2007). Genetic and molecular pathogenesis of mantle cell lymphoma: Perspectives for new targeted therapeutics. Nat. Rev. Cancer.

[32] Vincent-Fabert C., Fiancette R., Rouaud P., Baudet C., Truffinet V., Magnone V., Guillaudeau A., Cogne M., Dubus P., Denizot Y. (2012). A defect of the INK4-Cdk4 checkpoint and Myc collaborate in blastoid mantle cell lymphoma-like lymphoma formation in mice. Am. J. Pathol..

[33] Choe J.Y., Yun J.Y., Na H.Y., Huh J., Shin S.J., Kim H.J., Paik J.H., Kim Y.A., Nam S.J., Jeon Y.K. (2016). MYC overexpression correlates with MYC amplification or translocation, and is associated with poor prognosis in mantle cell lymphoma. Histopathology.

[34] Li Y., Bouchlaka M.N., Wolff J., Grindle K.M., Lu L., Qian S., Zhong X., Pflum N., Jobin P., Kahl B.S. (2016). FBXO10 deficiency and BTK activation upregulate BCL2 expression in mantle cell lymphoma. Oncogene.

[35] Yi S., Zou D., Li C., Zhong S., Chen W., Li Z., Xiong W., Liu W., Liu E., Cui R. (2015). High incidence of MYC and BCL2 abnormalities in mantle cell lymphoma, although only MYC abnormality predicts poor survival. Oncotarget.

[36] Bea S., Salaverria I., Armengol L., Pinyol M., Fernandez V., Hartmann E.M., Jares P., Amador V., Hernandez L., Navarro A. (2009). Uniparental disomies, homozygous deletions, amplifications, and target genes in mantle cell lymphoma revealed by integrative high-resolution whole-genome profiling. Blood.

[37] Pekarsky Y., Balatti V., Croce C.M. (2018). BCL2 and miR-15/16: From gene discovery to treatment. Cell Death Differ..

[38] Khoury J.D., Medeiros L.J., Rassidakis G.Z., McDonnell T.J., Abruzzo L.V., Lai R. (2003). Expression of Mcl-1 in mantle cell lymphoma is associated with high-grade morphology, a high proliferative state, and p53 overexpression. J. Pathol..

[39] Tagawa H., Karnan S., Suzuki R., Matsuo K., Zhang X., Ota A., Morishima Y., Nakamura S., Seto M. (2005). Genome-wide array-based CGH for mantle cell lymphoma: Identification of homozygous deletions of the proapoptotic gene BIM. Oncogene.

[40] Mestre-Escorihuela C., Rubio-Moscardo F., Richter J.A., Siebert R., Climent J., Fresquet V., Beltran E., Agirre X., Marugan I., Marin M. (2007). Homozygous deletions localize novel tumor suppressor genes in B-cell lymphomas. Blood.

[41] Katz S.G., Labelle J.L., Meng H., Valeriano R.P., Fisher J.K., Sun H., Rodig S.J., Kleinstein S.H., Walensky L.D. (2014). Mantle cell lymphoma in cyclin D1 transgenic mice with Bim-deficient B cells. Blood.

[42] Prukova D., Andera L., Nahacka Z., Karolova J., Svaton M., Klanova M., Havranek O., Soukup J., Svobodova K., Zemanova Z. (2019). Co-targeting of BCL2 with venetoclax and MCL1 with S63845 is synthetically lethal in vivo in relapsed mantle cell lymphoma. Clin. Cancer Res..

[43] Merolle M.I., Ahmed M., Nomie K., Wang M.L. (2018). The B cell receptor signaling pathway in mantle cell lymphoma. Oncotarget.

[44] Rao E., Jiang C., Ji M., Huang X., Iqbal J., Lenz G., Wright G., Staudt L.M., Zhao Y., McKeithan T.W. (2012). The miRNA-17 approximately 92 cluster mediates chemoresistance and enhances tumor growth in mantle cell lymphoma via PI3K/AKT pathway activation. Leukemia.

[45] Kahl B.S., Spurgeon S.E., Furman R.R., Flinn I.W., Coutre S.E., Brown J.R., Benson D.M., Byrd J.C., Peterman S., Cho Y. (2014). A phase 1 study of the PI3Kdelta inhibitor idelalisib in patients with relapsed/refractory mantle cell lymphoma (MCL). Blood.

[46] Thome M., Charton J.E., Pelzer C., Hailfinger S. (2010). Antigen receptor signaling to NF-kappaB via CARMA1, BCL10, and MALT1. Cold Spring Harb. Perspect. Biol..

[47] Zhang Q., Lenardo M.J., Baltimore D. (2017). 30 Years of NF-κB: A Blossoming of Relevance to Human Pathobiology. Cell.

[48] Yang Y., Kelly P., Shaffer A.L., Schmitz R., Yoo H.M., Liu X., Huang D.W., Webster D., Young R.M., Nakagawa M. (2016). Targeting Non-proteolytic Protein Ubiquitination for the Treatment of Diffuse Large B Cell Lymphoma. Cancer Cell.

[49] Keats J.J., Fonseca R., Chesi M., Schop R., Baker A., Chng W.J., Van Wier S., Tiedemann R., Shi C.X., Sebag M. (2007). Promiscuous mutations activate the noncanonical NF-κB pathway in multiple myeloma. Cancer Cell.

[50] Rossi D., Fangazio M., Rasi S., Vaisitti T., Monti S., Cresta S., Chiaretti S., Del Giudice I., Fabbri G., Bruscaggin A. (2012). Disruption of BIRC3 associates with fludarabine chemorefractoriness in TP53 wild-type chronic lymphocytic leukemia. Blood.

[51] Diop F., Moia R., Favini C., Spaccarotella E., De Paoli L., Bruscaggin A., Spina V., Terzi-di-Bergamo L., Arruga F., Tarantelli C. (2019). Biological and clinical implications of BIRC3 mutations in chronic lymphocytic leukemia. Haematologica.

[52] Dai B., Grau M., Juilland M., Klener P., Horing E., Molinsky J., Schimmack G., Aukema S.M., Hoster E., Vogt N. (2017). B-cell receptor-driven MALT1 activity regulates MYC signaling in mantle cell lymphoma. Blood.

[53] Kopan R., Ilagan M.X. (2009). The canonical Notch signaling pathway: Unfolding the activation mechanism. Cell.

[54] Inamdar A.A., Goy A., Ayoub N.M., Attia C., Oton L., Taruvai V., Costales M., Lin Y.T., Pecora A., Suh K.S. (2016). Mantle cell lymphoma in the era of precision medicine-diagnosis, biomarkers and therapeutic agents. Oncotarget.

[55] Swaroop A., Oyer J.A., Will C.M., Huang X., Yu W., Troche C., Bulic M., Durham B.H., Wen Q.J., Crispino J.D. (2019). An activating mutation of the NSD2 histone methyltransferase drives oncogenic reprogramming in acute lymphocytic leukemia. Oncogene.

[56] Zhang J., Lee Y.R., Dang F., Gan W., Menon A.V., Katon J.M., Hsu C.H., Asara J.M., Tibarewal P., Leslie N.R. (2019). PTEN Methylation by NSD2 Controls Cellular Sensitivity to DNA Damage. Cancer Discov..

[57] Jain P., Kanagal-Shamanna R., Zhang S., Ahmed M., Ghorab A., Zhang L., Ok C.Y., Li S., Hagemeister F., Zeng D. (2018). Long-term outcomes and mutation profiling of patients with mantle cell lymphoma (MCL) who discontinued ibrutinib. Br. J. Haematol..

[58] Shen Y., Morishita M., Lee D., Kim S., Lee T., Mevius D., Roh Y., di Luccio E. (2019). Identification of LEM-14 inhibitor of the oncoprotein NSD2. Biochem. Biophys. Res. Commun..

[59] Pasqualucci L., Trifonov V., Fabbri G., Ma J., Rossi D., Chiarenza A., Wells V.A., Grunn A., Messina M., Elliot O. (2011). Analysis of the coding genome of diffuse large B-cell lymphoma. Nat. Genet..

[60] Chen R., Frankel P., Popplewell L., Siddiqi T., Ruel N., Rotter A., Thomas S.H., Mott M., Nathwani N., Htut M. (2015). A phase II study of vorinostat and rituximab for treatment of newly diagnosed and relapsed/refractory indolent non-Hodgkin lymphoma. Haematologica.

[61] Oki Y., Buglio D., Fanale M., Fayad L., Copeland A., Romaguera J., Kwak L.W., Pro B., de Castro Faria S., Neelapu S. (2013). Phase I study of panobinostat plus everolimus in patients with relapsed or refractory lymphoma. Clin. Cancer Res..

[62] Evens A.M., Balasubramanian S., Vose J.M., Harb W., Gordon L.I., Langdon R., Sprague J., Sirisawad M., Mani C., Yue J. (2016). A Phase I/II Multicenter, Open-Label Study of the Oral Histone Deacetylase Inhibitor Abexinostat in Relapsed/Refractory Lymphoma. Clin. Cancer Res..

[63] Yazbeck V., Shafer D., Perkins E.B., Coppola D., Sokol L., Richards K.L., Shea T., Ruan J., Parekh S., Strair R. (2018). A Phase II Trial of Bortezomib and Vorinostat in Mantle Cell Lymphoma and Diffuse Large B-cell Lymphoma. Clin. LymphomaMyeloma Leuk..

[64] Shin D.Y., Kim S.J., Yoon D.H., Park Y., Kong J.H., Kim J.A., Kim B.S., Kim H.J., Won J.H., Park S.K. (2016). Results of a phase II study of vorinostat in combination with intravenous fludarabine, mitoxantrone, and dexamethasone in patients with relapsed or refractory mantle cell lymphoma: An interim analysis. Cancer Chemother. Pharmacol..

[65] Agarwal R., Chan Y.-C., Tam C.S., Hunter T., Vassiliadis D., Teh C.E., Thijssen R., Yeh P., Wong S.Q., Ftouni S. (2019). Dynamic molecular monitoring reveals that SWI–SNF mutations mediate resistance to ibrutinib plus venetoclax in mantle cell lymphoma. Nat. Med..

[66] Royo C., Navarro A., Clot G., Salaverria I., Gine E., Jares P., Colomer D., Wiestner A., Wilson W.H., Vegliante M.C. (2012). Non-nodal type of mantle cell lymphoma is a specific biological and clinical subgroup of the disease. Leukemia.

[67] Queiros A.C., Beekman R., Vilarrasa-Blasi R., Duran-Ferrer M., Clot G., Merkel A., Raineri E., Russinol N., Castellano G., Bea S. (2016). Decoding the DNA Methylome of Mantle Cell Lymphoma in the Light of the Entire B Cell Lineage. Cancer Cell.

[68] Vegliante M.C., Palomero J., Perez-Galan P., Roue G., Castellano G., Navarro A., Clot G., Moros A., Suarez-Cisneros H., Bea S. (2013). SOX11 regulates PAX5 expression and blocks terminal B-cell differentiation in aggressive mantle cell lymphoma. Blood.

[69] Ribera-Cortada I., Martinez D., Amador V., Royo C., Navarro A., Bea S., Gine E., de Leval L., Serrano S., Wotherspoon A. (2015). Plasma cell and terminal B-cell differentiation in mantle cell lymphoma mainly occur in the SOX11-negative subtype. Mod. Pathol..

[70] Kuo P.Y., Jatiani S.S., Rahman A.H., Edwards D., Jiang Z., Ahr K., Perumal D., Leshchenko V.V., Brody J., Shaknovich R. (2018). SOX11 augments BCR signaling to drive MCL-like tumor development. Blood.

[71] Palomero J., Vegliante M.C., Rodriguez M.L., Eguileor A., Castellano G., Planas-Rigol E., Jares P., Ribera-Cortada I., Cid M.C., Campo E. (2014). SOX11 promotes tumor angiogenesis through transcriptional regulation of PDGFA in mantle cell lymphoma. Blood.

[72] Balsas P., Palomero J., Eguileor A., Rodriguez M.L., Vegliante M.C., Planas-Rigol E., Sureda-Gomez M., Cid M.C., Campo E., Amador V. (2017). SOX11 promotes tumor protective microenvironment interactions through CXCR4 and FAK regulation in mantle cell lymphoma. Blood.

[73] Hoster E., Dreyling M., Klapper W., Gisselbrecht C., van Hoof A., Kluin-Nelemans H.C., Pfreundschuh M., Reiser M., Metzner B., Einsele H. (2008). A new prognostic index (MIPI) for patients with advanced-stage mantle cell lymphoma. Blood.

[74] Hoster E., Klapper W., Hermine O., Kluin-Nelemans H.C., Walewski J., van Hoof A., Trneny M., Geisler C.H., Di Raimondo F., Szymczyk M. (2014). Confirmation of the mantle-cell lymphoma International Prognostic Index in randomized trials of the European Mantle-Cell Lymphoma Network. J. Clin. Oncol..

[75] Greenwell I.B., Staton A.D., Lee M.J., Switchenko J.M., Saxe D.F., Maly J.J., Blum K.A., Grover N.S., Mathews S.P., Gordon M.J. (2018). Complex karyotype in patients with mantle cell lymphoma predicts inferior survival and poor response to intensive induction therapy. Cancer.

[76] Sarkozy C., Terre C., Jardin F., Radford I., Roche-Lestienne C., Penther D., Bastard C., Rigaudeau S., Pilorge S., Morschhauser F. (2014). Complex karyotype in mantle cell lymphoma is a strong prognostic factor for the time to treatment and overall survival, independent of the MCL international prognostic index. Genes Chromosom. Cancer.

[77] Determann O., Hoster E., Ott G., Wolfram Bernd H., Loddenkemper C., Leo Hansmann M., Barth T.E., Unterhalt M., Hiddemann W., Dreyling M. (2008). Ki-67 predicts outcome in advanced-stage mantle cell lymphoma patients treated with anti-CD20 immunochemotherapy: Results from randomized trials of the European MCL Network and the German Low Grade Lymphoma Study Group. Blood.

[78] Hoster E., Rosenwald A., Berger F., Bernd H.W., Hartmann S., Loddenkemper C., Barth T.F., Brousse N., Pileri S., Rymkiewicz G. (2016). Prognostic Value of Ki-67 Index, Cytology, and Growth Pattern in Mantle-Cell Lymphoma: Results From Randomized Trials of the European Mantle Cell Lymphoma Network. J. Clin. Oncol..

[79] Dreyling M., Klapper W., Rule S. (2018). Blastoid and pleomorphic mantle cell lymphoma: Still a diagnostic and therapeutic challenge!. Blood.

[80] Ruan J., Martin P., Shah B., Schuster S.J., Smith S.M., Furman R.R., Christos P., Rodriguez A., Svoboda J., Lewis J. (2015). Lenalidomide plus Rituximab as Initial Treatment for Mantle-Cell Lymphoma. N. Engl. J. Med..

[81] Kumar A., Ying Z., Alperovich A., Dogan A., Hamlin P., Moskowitz C., Pichardo J., Portlock C., Sha F., Zelenetz A.D. (2019). Clinical presentation determines selection of patients for initial observation in mantle cell lymphoma. Haematologica.

[82] Hermine O., Hoster E., Walewski J., Bosly A., Stilgenbauer S., Thieblemont C., Szymczyk M., Bouabdallah R., Kneba M., Hallek M. (2016). Addition of high-dose cytarabine to immunochemotherapy before autologous stem-cell transplantation in patients aged 65 years or younger with mantle cell lymphoma (MCL Younger): A randomised, open-label, phase 3 trial of the European Mantle Cell Lymphoma Network. Lancet.

[83] Geisler C.H., Kolstad A., Laurell A., Andersen N.S., Pedersen L.B., Jerkeman M., Eriksson M., Nordstrom M., Kimby E., Boesen A.M. (2008). Long-term progression-free survival of mantle cell lymphoma after intensive front-line immunochemotherapy with in vivo-purged stem cell rescue: A nonrandomized phase 2 multicenter study by the Nordic Lymphoma Group. Blood.

[84] Geisler C.H., Kolstad A., Laurell A., Jerkeman M., Raty R., Andersen N.S., Pedersen L.B., Eriksson M., Nordstrom M., Kimby E. (2012). Nordic MCL2 trial update: Six-year follow-up after intensive immunochemotherapy for untreated mantle cell lymphoma followed by BEAM or BEAC + autologous stem-cell support: Still very long survival but late relapses do occur. Br. J. Haematol..

[85] Lenz G., Dreyling M., Hoster E., Wormann B., Duhrsen U., Metzner B., Eimermacher H., Neubauer A., Wandt H., Steinhauer H. (2005). Immunochemotherapy with rituximab and cyclophosphamide, doxorubicin, vincristine, and prednisone significantly improves response and time to treatment failure, but not long-term outcome in patients with previously untreated mantle cell lymphoma: Results of a prospective randomized trial of the German Low Grade Lymphoma Study Group (GLSG). J. Clin. Oncol..

[86] Gerson J.N., Barta S.K. (2019). Mantle Cell Lymphoma: Which Patients Should We Transplant?. Curr. Hematol. Malig. Rep..

[87] Le Gouill S., Thieblemont C., Oberic L., Moreau A., Bouabdallah K., Dartigeas C., Damaj G., Gastinne T., Ribrag V., Feugier P. (2017). Rituximab after Autologous Stem-Cell Transplantation in Mantle-Cell Lymphoma. N. Engl. J. Med..

[88] Dietrich S., Weidle J., Rieger M., Meissner J., Radujkovic A., Ho A.D., Dreger P., Witzens-Harig M. (2014). Rituximab maintenance therapy after autologous stem cell transplantation prolongs progression-free survival in patients with mantle cell lymphoma. Leukemia.

[89] Graf S.A., Stevenson P.A., Holmberg L.A., Till B.G., Press O.W., Chauncey T.R., Smith S.D., Philip M., Orozco J.J., Shustov A.R. (2015). Maintenance rituximab after autologous stem cell transplantation in patients with mantle cell lymphoma. Ann. Oncol..

[90] Mei M.G., Cao T.M., Chen L., Song J.Y., Siddiqi T., Cai J.L., Farol L.T., Al Malki M.M., Salhotra A., Aldoss I. (2017). Long-Term Results of High-Dose Therapy and Autologous Stem Cell Transplantation for Mantle Cell Lymphoma: Effectiveness of Maintenance Rituximab. Biol. Blood Marrow Transplant. J. Am. Soc. Blood Marrow Transplant..

[91] Kumar A., Sha F., Toure A., Dogan A., Ni A., Batlevi C.L., Palomba M.L.M., Portlock C., Straus D.J., Noy A. (2019). Patterns of survival in patients with recurrent mantle cell lymphoma in the modern era: Progressive shortening in response duration and survival after each relapse. Blood Cancer J..

[92] Gerson J.N., Handorf E., Villa D., Gerrie A.S., Chapani P., Li S., Medeiros L.J., Wang M.I., Cohen J.B., Calzada O. (2019). Survival Outcomes of Younger Patients With Mantle Cell Lymphoma Treated in the Rituximab Era. J. Clin. Oncol..

[93] Visco C., Finotto S., Zambello R., Paolini R., Menin A., Zanotti R., Zaja F., Semenzato G., Pizzolo G., D’Amore E.S. (2013). Combination of rituximab, bendamustine, and cytarabine for patients with mantle-cell non-Hodgkin lymphoma ineligible for intensive regimens or autologous transplantation. J. Clin. Oncol..

[94] Rummel M.J., Niederle N., Maschmeyer G., Banat G.A., von Grunhagen U., Losem C., Kofahl-Krause D., Heil G., Welslau M., Balser C. (2013). Bendamustine plus rituximab versus CHOP plus rituximab as first-line treatment for patients with indolent and mantle-cell lymphomas: An open-label, multicentre, randomised, phase 3 non-inferiority trial. Lancet.

[95] Robak T., Jin J., Pylypenko H., Verhoef G., Siritanaratkul N., Drach J., Raderer M., Mayer J., Pereira J., Tumyan G. (2018). Frontline bortezomib, rituximab, cyclophosphamide, doxorubicin, and prednisone (VR-CAP) versus rituximab, cyclophosphamide, doxorubicin, vincristine, and prednisone (R-CHOP) in transplantation-ineligible patients with newly diagnosed mantle cell lymphoma: Final overall survival results of a randomised, open-label, phase 3 study. Lancet. Oncol..

[96] Kluin-Nelemans H.C., Hoster E., Hermine O., Walewski J., Trneny M., Geisler C.H., Stilgenbauer S., Thieblemont C., Vehling-Kaiser U., Doorduijn J.K. (2012). Treatment of older patients with mantle-cell lymphoma. N. Engl. J. Med..

[97] Visco C., Chiappella A., Nassi L., Patti C., Ferrero S., Barbero D., Evangelista A., Spina M., Molinari A., Rigacci L. (2017). Rituximab, bendamustine, and low-dose cytarabine as induction therapy in elderly patients with mantle cell lymphoma: A multicentre, phase 2 trial from Fondazione Italiana Linfomi. Lancet Haematol..

[98] Obr A., Prochazka V., Papajik T., Klener P., Janikova A., Salek D., Belada D., Pytlik R., Sykorova A., Mocikova H. (2019). Maintenance rituximab in newly diagnosed mantle cell lymphoma patients: A real world analysis from the Czech lymphoma study group registry(dagger). Leuk. Lymphoma.

[99] Chen R.W., Palmer J.M., Tomassetti S., Popplewell L.L., Alluin J., Chomchan P., Nademanee A.P., Siddiqi T., Tsai N.C., Chen L. (2018). Multi-center phase II trial of bortezomib and rituximab maintenance combination therapy in patients with mantle cell lymphoma after consolidative autologous stem cell transplantation. J. Hematol. Oncol..

[100] Till B.G. (2018). Maintenance Therapy in Diffuse Large B Cell Lymphoma and Mantle Cell Lymphoma. Curr. Treat. Options Oncol..

[101] Klener P., Fronkova E., Kalinova M., Belada D., Forsterova K., Pytlik R., Blahovcova P., Simkovic M., Salek D., Mocikova H. (2018). Potential loss of prognostic significance of minimal residual disease assessment after R-CHOP-based induction in elderly patients with mantle cell lymphoma in the era of rituximab maintenance. Hematol. Oncol..

[102] Visco C., Tisi M.C., Evangelista A., Di Rocco A., Zoellner A.K., Zilioli V.R., Hohaus S., Sciarra R., Re A., Tecchio C. (2019). Time to progression of mantle cell lymphoma after high-dose cytarabine-based regimens defines patients risk for death. Br. J. Haematol..

[103] Pott C., Hoster E., Delfau-Larue M.H., Beldjord K., Bottcher S., Asnafi V., Plonquet A., Siebert R., Callet-Bauchu E., Andersen N. (2010). Molecular remission is an independent predictor of clinical outcome in patients with mantle cell lymphoma after combined immunochemotherapy: A European MCL intergroup study. Blood.

[104] Andersen N.S., Pedersen L.B., Laurell A., Elonen E., Kolstad A., Boesen A.M., Pedersen L.M., Lauritzsen G.F., Ekanger R., Nilsson-Ehle H. (2009). Pre-emptive treatment with rituximab of molecular relapse after autologous stem cell transplantation in mantle cell lymphoma. J. Clin. Oncol..

[105] Pott C., Bruggemann M., Ritgen M., van der Velden V.H.J., van Dongen J.J.M., Kneba M. (2019). MRD Detection in B-Cell Non-Hodgkin Lymphomas Using Ig Gene Rearrangements and Chromosomal Translocations as Targets for Real-Time Quantitative PCR. Methods Mol. Biol. (CliftonN.J.).

[106] Smith A., Roman E., Appleton S., Howell D., Johnson R., Burton C., Patmore R. (2018). Impact of novel therapies for mantle cell lymphoma in the real world setting: A report from the UK’s Haematological Malignancy Research Network (HMRN). Br. J. Haematol..

[107] Epperla N., Hamadani M., Fenske T.S., Costa L.J. (2018). Incidence and survival trends in mantle cell lymphoma. Br. J. Haematol..

[108] Klanova M., Lorkova L., Vit O., Maswabi B., Molinsky J., Pospisilova J., Vockova P., Mavis C., Lateckova L., Kulvait V. (2014). Downregulation of deoxycytidine kinase in cytarabine-resistant mantle cell lymphoma cells confers cross-resistance to nucleoside analogs gemcitabine, fludarabine and cladribine, but not to other classes of anti-lymphoma agents. Mol. Cancer.

[109] Wang Y., Zhang L.L., Champlin R.E., Wang M.L. (2015). Targeting Bruton’s tyrosine kinase with ibrutinib in B-cell malignancies. Clin. Pharmacol. Ther..

[110] Wang M., Rule S., Zinzani P.L., Goy A., Casasnovas O., Smith S.D., Damaj G., Doorduijn J., Lamy T., Morschhauser F. (2018). Acalabrutinib in relapsed or refractory mantle cell lymphoma (ACE-LY-004): A single-arm, multicentre, phase 2 trial. Lancet.

[111] Wang M.L., Rule S., Martin P., Goy A., Auer R., Kahl B.S., Jurczak W., Advani R.H., Romaguera J.E., Williams M.E. (2013). Targeting BTK with ibrutinib in relapsed or refractory mantle-cell lymphoma. N. Engl. J. Med..

[112] Dreyling M., Jurczak W., Jerkeman M., Silva R.S., Rusconi C., Trneny M., Offner F., Caballero D., Joao C., Witzens-Harig M. (2016). Ibrutinib versus temsirolimus in patients with relapsed or refractory mantle-cell lymphoma: An international, randomised, open-label, phase 3 study. Lancet.

[113] Martin P., Maddocks K., Leonard J.P., Ruan J., Goy A., Wagner-Johnston N., Rule S., Advani R., Iberri D., Phillips T. (2016). Postibrutinib outcomes in patients with mantle cell lymphoma. Blood.

[114] Cheah C.Y., Chihara D., Romaguera J.E., Fowler N.H., Seymour J.F., Hagemeister F.B., Champlin R.E., Wang M.L. (2015). Patients with mantle cell lymphoma failing ibrutinib are unlikely to respond to salvage chemotherapy and have poor outcomes. Ann. Oncol..

[115] Epperla N., Hamadani M., Cashen A.F., Ahn K.W., Oak E., Kanate A.S., Calzada O., Cohen J.B., Farmer L., Ghosh N. (2017). Predictive factors and outcomes for ibrutinib therapy in relapsed/refractory mantle cell lymphoma-a “real world” study. Hematol. Oncol..

[116] Dreger P., Michallet M., Bosman P., Dietrich S., Sobh M., Boumendil A., Nagler A., Scheid C., Cornelissen J., Niederwieser D. (2019). Ibrutinib for bridging to allogeneic hematopoietic cell transplantation in patients with chronic lymphocytic leukemia or mantle cell lymphoma: A study by the EBMT Chronic Malignancies and Lymphoma Working Parties. Bone Marrow Transplant..

[117] Rule S., Cook G., Russell N.H., Hunter A., Robinson S., Morley N., Sureda A., Patrick P., Clifton-Hadley L., Adedayo T. (2019). Allogeneic stem cell transplantation as part of front line therapy for Mantle cell lymphoma. Br. J. Haematol..

[118] Ma J., Lu P., Guo A., Cheng S., Zong H., Martin P., Coleman M., Wang Y.L. (2014). Characterization of ibrutinib-sensitive and -resistant mantle lymphoma cells. Br. J. Haematol..

[119] Hershkovitz-Rokah O., Pulver D., Lenz G., Shpilberg O. (2018). Ibrutinib resistance in mantle cell lymphoma: Clinical, molecular and treatment aspects. Br. J. Haematol..

[120] Chiron D., Di Liberto M., Martin P., Huang X., Sharman J., Blecua P., Mathew S., Vijay P., Eng K., Ali S. (2014). Cell-cycle reprogramming for PI3K inhibition overrides a relapse-specific C481S BTK mutation revealed by longitudinal functional genomics in mantle cell lymphoma. Cancer Discov..

[121] Zhao X., Lwin T., Silva A., Shah B., Tao J., Fang B., Zhang L., Fu K., Bi C., Li J. (2017). Unification of de novo and acquired ibrutinib resistance in mantle cell lymphoma. Nat. Commun..

[122] Compagno M., Wang Q., Pighi C., Cheong T.C., Meng F.L., Poggio T., Yeap L.S., Karaca E., Blasco R.B., Langellotto F. (2017). Phosphatidylinositol 3-kinase delta blockade increases genomic instability in B cells. Nature.

[123] Martin P., Bartlett N.L., Blum K.A., Park S., Maddocks K., Ruan J., Ridling L., Dittus C., Chen Z., Huang X. (2019). A phase 1 trial of ibrutinib plus palbociclib in previously treated mantle cell lymphoma. Blood.

[124] Wang M.L., Lee H., Chuang H., Wagner-Bartak N., Hagemeister F., Westin J., Fayad L., Samaniego F., Turturro F., Oki Y. (2016). Ibrutinib in combination with rituximab in relapsed or refractory mantle cell lymphoma: A single-centre, open-label, phase 2 trial. Lancet. Oncol..

[125] Tam C.S., Anderson M.A., Pott C., Agarwal R., Handunnetti S., Hicks R.J., Burbury K., Turner G., Di Iulio J., Bressel M. (2018). Ibrutinib plus Venetoclax for the Treatment of Mantle-Cell Lymphoma. N. Engl. J. Med..

[126] Robak P., Robak T. (2019). Bortezomib for the Treatment of Hematologic Malignancies: 15 Years Later. Drugs RD.

[127] Fisher R.I., Bernstein S.H., Kahl B.S., Djulbegovic B., Robertson M.J., de Vos S., Epner E., Krishnan A., Leonard J.P., Lonial S. (2006). Multicenter phase II study of bortezomib in patients with relapsed or refractory mantle cell lymphoma. J. Clin. Oncol..

[128] Lamm W., Kaufmann H., Raderer M., Hoffmann M., Chott A., Zielinski C., Drach J. (2011). Bortezomib combined with rituximab and dexamethasone is an active regimen for patients with relapsed and chemotherapy-refractory mantle cell lymphoma. Haematologica.

[129] Friedberg J.W., Vose J.M., Kelly J.L., Young F., Bernstein S.H., Peterson D., Rich L., Blumel S., Proia N.K., Liesveld J. (2011). The combination of bendamustine, bortezomib, and rituximab for patients with relapsed/refractory indolent and mantle cell non-Hodgkin lymphoma. Blood.

[130] Zinzani P.L., Vose J.M., Czuczman M.S., Reeder C.B., Haioun C., Polikoff J., Tilly H., Zhang L., Prandi K., Li J. (2013). Long-term follow-up of lenalidomide in relapsed/refractory mantle cell lymphoma: Subset analysis of the NHL-003 study. Ann. Oncol..

[131] Goy A., Sinha R., Williams M.E., Kalayoglu Besisik S., Drach J., Ramchandren R., Zhang L., Cicero S., Fu T., Witzig T.E. (2013). Single-agent lenalidomide in patients with mantle-cell lymphoma who relapsed or progressed after or were refractory to bortezomib: Phase II MCL-001 (EMERGE) study. J. Clin. Oncol..

[132] Trneny M., Lamy T., Walewski J., Belada D., Mayer J., Radford J., Jurczak W., Morschhauser F., Alexeeva J., Rule S. (2016). Lenalidomide versus investigator’s choice in relapsed or refractory mantle cell lymphoma (MCL-002; SPRINT): A phase 2, randomised, multicentre trial. Lancet Oncol..

[133] Hagner P.R., Chiu H., Ortiz M., Apollonio B., Wang M., Couto S., Waldman M.F., Flynt E., Ramsay A.G., Trotter M. (2017). Activity of lenalidomide in mantle cell lymphoma can be explained by NK cell-mediated cytotoxicity. Br. J. Haematol..

[134] Hess G., Herbrecht R., Romaguera J., Verhoef G., Crump M., Gisselbrecht C., Laurell A., Offner F., Strahs A., Berkenblit A. (2009). Phase III study to evaluate temsirolimus compared with investigator’s choice therapy for the treatment of relapsed or refractory mantle cell lymphoma. J. Clin. Oncol..

[135] Davids M.S., Roberts A.W., Seymour J.F., Pagel J.M., Kahl B.S., Wierda W.G., Puvvada S., Kipps T.J., Anderson M.A., Salem A.H. (2017). Phase I First-in-Human Study of Venetoclax in Patients with Relapsed or Refractory Non-Hodgkin Lymphoma. J. Clin. Oncol..

[136] Eyre T.A., Walter H.S., Iyengar S., Follows G., Cross M., Fox C.P., Hodson A., Coats J., Narat S., Morley N. (2019). Efficacy of venetoclax monotherapy in patients with relapsed, refractory mantle cell lymphoma after Bruton tyrosine kinase inhibitor therapy. Haematologica.

[137] Andorsky D.J., Kolibaba K.S., Assouline S., Forero-Torres A., Jones V., Klein L.M., Patel-Donnelly D., Smith M., Ye W., Shi W. (2019). An open-label phase 2 trial of entospletinib in indolent non-Hodgkin lymphoma and mantle cell lymphoma. Br. J. Haematol..

[138] Morschhauser F., Seymour J.F., Kluin-Nelemans H.C., Grigg A., Wolf M., Pfreundschuh M., Tilly H., Raemaekers J., van’t Veer M.B., Milpied N. (2008). A phase II study of enzastaurin, a protein kinase C beta inhibitor, in patients with relapsed or refractory mantle cell lymphoma. Ann. Oncol..

[139] Xu-Monette Z.Y., Zhou J., Young K.H. (2018). PD-1 expression and clinical PD-1 blockade in B-cell lymphomas. Blood.

[140] Levin A., Shah N.N. (2019). Chimeric antigen receptor modified T cell therapy in B cell non-Hodgkin lymphomas. Am. J. Hematol..

[141] Montico B., Lapenta C., Ravo M., Martorelli D., Muraro E., Zeng B., Comaro E., Spada M., Donati S., Santini S.M. (2017). Exploiting a new strategy to induce immunogenic cell death to improve dendritic cell-based vaccines for lymphoma immunotherapy. Oncoimmunology.

[142] Cox M.C., Castiello L., Mattei M., Santodonato L., D’Agostino G., Muraro E., Martorelli D., Lapenta C., Di Napoli A., Di Landro F. (2019). Clinical and Antitumor Immune Responses in Relapsed/Refractory Follicular Lymphoma Patients after Intranodal Injections of IFNalpha-Dendritic Cells and Rituximab. Clin. Cancer Res..

